# Cerebrospinal fluid infiltration of primary cutaneous gamma delta T‐cell lymphoma

**DOI:** 10.1002/jha2.329

**Published:** 2021-11-24

**Authors:** Yoshikazu Hori, Yu Aruga, Chiaki Ikeda, Akiko Miyagi Maeshima, Koji Izutsu, Hiromichi Matsushita

**Affiliations:** ^1^ Department of Haematology, National Cancer Centre Hospital Tokyo Japan; ^2^ Department of Laboratory Medicine, National Cancer Centre Hospital Tokyo Japan; ^3^ Department of Pathology National Cancer Centre Hospital Tokyo Japan

A 63‐year‐old man recognised a growing cutaneous elevation on his left palm that reached 6 cm in diameter within 2 months. A histological examination revealed a diagnosis of primary cutaneous gamma delta T‐cell lymphoma (PCGD‐TCL) (Figure 1, **panels A** and **B**). Positron emission tomography showed tumour involvement in the left palm, left arm, left axillary and subclavian lymph nodes, and prostate gland, suggesting his disease was clinical stage IV. After receiving four cycles of CHOP (cyclophosphamide, doxorubicin, vincristine, and prednisolone), he suddenly developed left facial and oculomotor nerve palsy. Gadolinium‐enhanced magnetic resonance imaging showed no brain lesion. However, cerebrospinal fluid (CSF) pleocytosis (87 cells/μl) was observed, accompanied by abnormal lymphoid cells that were medium to large in size with a nucleus harbouring fine chromatin formation and multiple nucleolus as well as an irregular cytoplasm contour (Figure 1, **panel C**). The cells were positive for CD3, CD5 and TCRγδ but not CD4, CD7 or CD8 (Figure 1, **panel D**, orange), demonstrating CSF infiltration of PCGD‐TCL.

**FIGURE 1:**
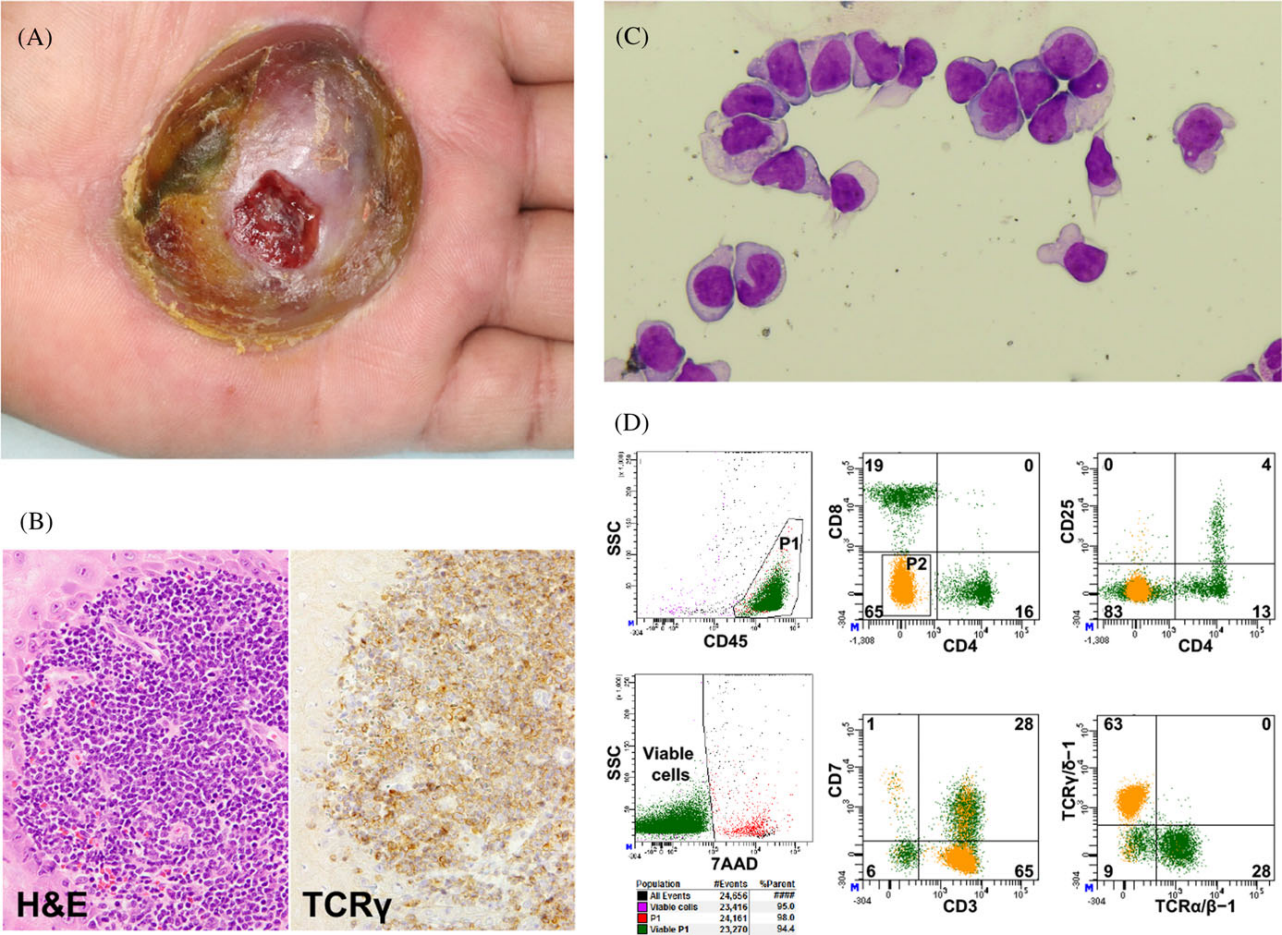
Cerebrospinal fluid infiltration of primary cutaneous gamma‐delta T‐cell lymphoma. (A) The macroscopic appearance of the primary cutaneous lesion on the left palm. (B) The histopathological findings of the cutaneous lesion. Left panel: H&E stain, 400×. Right panel: anti‐TCRγ stain, 400×. (C) The lymphoma cells infiltrated the cerebrospinal fluid (May–Grünwald–Giemsa stain). (D) A flow cytometric analysis of the lymphoma cells in the cerebrospinal fluid. CD4^–^ CD8^–^ (double‐negative) cells are shown in orange

PCGD‐TCL, a rare type of aggressive T‐cell lymphoma, often presents as generalised skin lesions on the extremities. Infiltration of the CSF, as well as the lymph nodes, spleen and bone marrow, is uncommon.

## AUTHOR CONTRIBUTIONS

Yoshikazu Hori managed the case and wrote the manuscript. Yu Aruga and Chiaki Ikeda performed the laboratory analyses. Akiko Miyagi Maeshima performed the histopathological and immunohistochemical analyses and reviewed the manuscript critically. Koji Izutsu diagnosed and managed the case and reviewed the manuscript critically. Hiromichi Matsushita wrote the manuscript and approved the submission of the final version.

## CONFLICT OF INTEREST

The authors declare no conflict of interest.

